# Supplementary material for: Multiscale modeling of human cerebrovasculature: A hybrid approach using image-based geometry and a mathematical algorithm
Source: PLoS Comput Biol. 2020 Jun 22;16(6):e1007943. doi: 10.1371/journal.pcbi.1007943 (PMC7332106; doi:10.1371/journal.pcbi.1007943)
Supplement: S1 Supplement — (PDF) [file pcbi.1007943.s001.pdf]

S1 Supplement: Validation of the vascular generation algorithm

The present vascular generation algorithm simplifies the original CCO algorithm, where the structural optimization is not taken into account and only four bifurcation patterns are considered in the geometric optimization. Moreover, we assume the wall shear stresses are equivalent everywhere, and that they easily determine the edge radii as the ratio of the flowrate to the root edge. We investigate the effect of these simplifications and modifications on the reconstructed vascular geometries. Note that we do not introduce the idea of the subregion, meaning the terminal vertices are randomly determined in the analysis domain.

We perform the same numerical example, in which vascular trees are generated from a single root in the 3-D domain. In this test, we set the reconstruction parameters: the root radius is 2.4 mm, the number of terminal ends 4000, and the perfusion domain has a volume of 100 cm^3. Here, we assume that all the terminal ends have the same flowrate, that is, the radius of any edge is determined by the ratio of the number of terminal ends of the focusing edge to that of the root edge. Again, the present model reflects the root radius by the constraint of the equivalent condition of the edge-wise wall shear stress, and thus the absolute value of the flowrate is not needed.

Fig S1.1 shows a transition of the mean diameter and standard deviation with respect to the bifurcation level from the root edge. The present result agrees well with both the original CCO algorithm and the measurement data available in the literature [1].

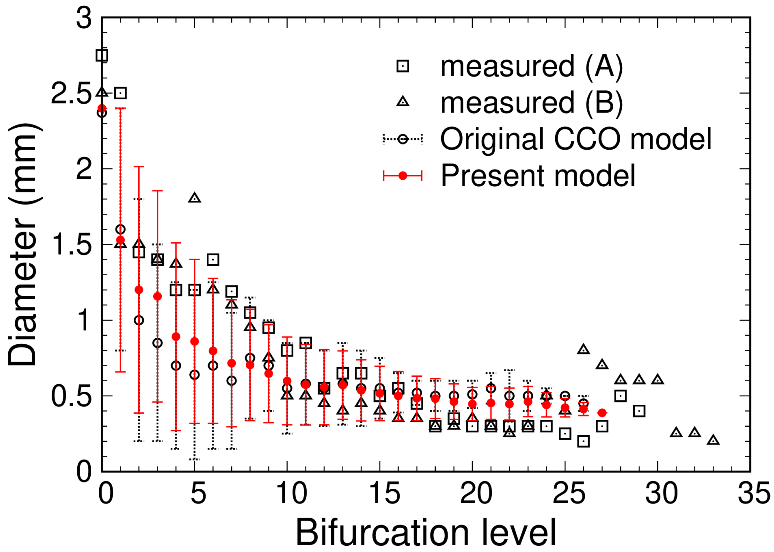

Fig S1.1. Comparison of the relationship between vascular diameter and bifurcation level given by the present model, CCO model, and measurements available in the literature [1].

References

[1] Karch R, Neumann F, Neumann M, Schreiner W. A three-dimensional model for arterial tree representation, generated by constrained constructive optimization. Comput Biol Med. 1999;29:19–38.
