# Supplementary material for: Multiscale modeling of human cerebrovasculature: A hybrid approach using image-based geometry and a mathematical algorithm
Source: PLoS Comput Biol. 2020 Jun 22;16(6):e1007943. doi: 10.1371/journal.pcbi.1007943 (PMC7332106; doi:10.1371/journal.pcbi.1007943)
Supplement: Attachment — Submitted filename: PLoSCompBIoCerebralNetworksLi.pdf [file pcbi.1007943.s002.pdf]

**Hybrid modeling of whole-scale cerebrovasculature based on personalized morphometry and mathematical algorithm**

By: Ii, Kitade, Ishida, Imai, Watanabe, and Wada

**General comments:**
While the topic of this study is fascinating the manuscript is very difficult to read, mainly due to the poor organization. Before being ready for publication I suggest that authors do the following:

1: Streamline your introduction to clearly describe what are the objectives of the paper – to generate a vascular network for the brain. The introduction to the current version of the manuscript misleads the reader thinking this is a paper on cerebral autoregulation, which it is not.

2: In the beginning of the methods section include a figure and clearly discuss the components included in the workflow. Include A: Generation of large vessel network from CT data – this section is almost absent in the current study. B: Generation of small vessel arterial and venous networks. Within the latter section, again clearly note components including a) prediction of vessel radius. I do not understand why this is determined from flow rather than using information from the image. b) clearly describe the optimality principle used to generate the network (it is still not clear to me what that is). c) describe network generation (this is currently done but it is hard to read) – it would help if authors had described the process in an algorithm environment. C: Discuss how generated networks compare to data, here include a thorough discussion of what data is available and how this data can be used for model validation.

3: The results and discussion sections are reasonable with the exception that they lack more clear description of how results found in this study compare to data.

**Specific comments:**
**Introduction –** Remove discussion of CFD and cerebral autoregulation – this study is about network generation informed by imaging data. Instead discuss literature on this topic.
The last paragraph of the introduction should clearly outline the objectives o this study. TO design an algorithm to generate networks linking large vessels extracted from CT images to small vessels generated using the proposed algorithm. The goal is to generate a network that fills volume best possible (I think).

**Methods –** The methods section should start with a figure clearly outlining the workflow. I would restructure the section starting with the sub-section discussing generation of large vessels from the CT images – this is currently described on page 8, but in a very short paragraph with too few details. This first section should refer to Fig. 4a and b with a clearer description of how each flow region are determined. Also discuss how other information available from the images are used, e.g. what is the total flow and how is it measured, how is data used to validate that the generated networks are correct.

Before in detail describing how the graphs representing the small vessels are generated include an algorithm highlighting the steps used to generate these graphs. Here it would be useful to describe differences between arterial and venous trees.

Section page 5 “Determination of edge radii on vascular trees”.
Line 125: This section starts by discussing fluid mechanics principles noting that fluid dynamics is assuming ideal conditions. What does that mean? Please specify in detail.

Please specify how flow is obtained from 4D-CT images – needed to understand why the radius is predicted as a function of flow – why not use information on radius from the images?

The paragraph on page 6 lines 160-172 is difficult to read – so the vessel radii depends on flow, but only on the size and number of terminal ends? But equation 13 states that the radii are determined from the flow. This is confusing. What role does the data play? Vessels in the images have radii – why not use these to inform the network generation?

Page 6 – “generation of a new bifurcation”. This paragraph mentions that the objective is to minimize volume – that information should have been introduced much earlier.

Page 7 – multilevel reconstruction. Are the networks generated starting at the root reaching a specific vascular subregion or are they just branching from the root until the entire domain is filled? That is not clear. Also, it is not clear if network construction is continued until a total number of vessels are included or if it is terminated when the vessels reach a certain size.

Page 8 – Table 1 mostly summarizes results and should therefore be referenced in the results section. Maybe the paragraph on “model parameters” really should be moved to the results section.

Page 9 – The first paragraph on page 9 discuss generation of arterial and venous trees and discuss that to include vascular variation each terminal subregion does not have the same number of vessels, this is also illustrated in Fig. 1, yet in Fig.6 authors note that the network shown here has exactly two arterial vessels and one vein – this is contradicting.

Page 10 – discuss results shown in Fig. 7 – it would be nice if authors have shown from where in (a) the enlarged insert shown in (b) is taken. Also, here authors do include (c) values from measurements. Are these from the same data, if so in the “data” part of the methods section, that I think should be placed first, authors should describe how this statistic was gathered.

Page 11 – Authors discuss ordering, using Strahler’s method – but the method is not given in the methods section – please add that. On Figure 8 it would be useful to superimpose values from literature to visualize how well the generated network match measurements, even if they are not from human networks.

Page 12 – Vascular territories – do the networks generated encode specific vascular territories a priori or do they emerge as part of the branching strategy. Please elaborate.

Page 12 – line 450. One of the Fig is referenced as ??.

Page 14 – Either in addition to Fig. 11 or as a new figure – it would be interesting if authors could elaborate on the effect on brain shape, e.g. by showing simulations from brains of different shapes, this could be expanded to show modification in brains with tumors or infarcts.
