# Supplementary material for: Multiscale modeling of human cerebrovasculature: A hybrid approach using image-based geometry and a mathematical algorithm
Source: PLoS Comput Biol. 2020 Jun 22;16(6):e1007943. doi: 10.1371/journal.pcbi.1007943 (PMC7332106; doi:10.1371/journal.pcbi.1007943)
Supplement: Attachment — Submitted filename: Review.pdf [file pcbi.1007943.s003.pdf]

Title: **Hybrid modeling of whole-scale cerebrovasculature based on personalized morphometric and mathematical algorithm**

Summary:

This paper presents a multiscale model of the human cerebrovasculature, based on a hybrid image-based reconstruction and hierarchical modeling approach. At the first of three levels (LV0), realistic macrovasculature is obtained by reconstructing the major cerebral arteries and veins from segmented 3D CT angiography images. Next, the terminal edges of the arteries and veins from level LV0 are used as input for the hierarchical vascular structure modeled at level LV1 (down to 100 µm diameter) using a "multilevel region-confined" (MRC) algorithm. Finally, the terminal edges level LV1 are in turn used as inputs to the second level LV2 using the same MRC algorithm in confined-within network structures representing pial vessels with diameter less than 100µm. LV1 and LV2 constitute the superficial cortical vessels. Biological and morphological features such as vascular territories supplied by the major cerebral arteries or the influence of cortical folding are investigated.

Overview:

With the increasing interest in the role of the vascular component in cerebral aging and neurodegenerative diseases, there is a growing need for an accurate description of brain blood and cerebrospinal flows along with autoregulation mechanisms. The authors present an interesting approach to construct the human brain vasculature by combining real CT and MRI-based data for the macrovasculature down to 1mm diameter, and mathematical modeling for smaller vessels. This model includes both arterial and venous networks, which will be crucial for subsequent blood flow modeling. The use of image-based reconstruction of the microvasculature allows accounting for complex features in the vascular architecture, such as the Circle of Willis in the arterial network, and for geometrical constraints related to the brain shape, such as the hemispheres boundaries but also more local constraints related to gyri and sulci. Such geometry would be impossible to reproduce from mathematical models only. When it comes to smaller vessels, *in-vivo* imaging is limited in spatial resolution and accurate reconstructions can only rely on post mortem techniques such as histology, on limited parts of the brain. Mathematical modeling is thus needed to provide a more complete picture. In this sense, the authors provide a very useful tool that will undoubtedly serve as a strong basis for further blood flow analysis through modeling and simulations.

Overall, this paper is well written and nicely illustrated. The mathematical descriptions are clear and the results are compared, not only with results obtained with the reference CCO model, but with real data from the literature, among which some still stand as a reference in the field (e.g. Duvernoy). I nonetheless have a few general and some more specific questions below that I would appreciate to see clarified and eventually discussed.

Comments:

The cerebral vasculature goes beyond the surface of the cortex. As long as the microvasculature, which not only includes arterioles and venules but also capillaries, is not accounted for, I wouldn't call this a "whole-scale" model but rather a "multiscale" model. Or use something like "cerebral macrovasculature" instead of "cerebrovasculature" and "entire cerebral vasculature".

It is stated in the "Model limitations and future improvements" that the "present formulation presents the possibility to address several types of anastomoses based on information on vascular subregions…". Can the authors be more specific?

From my understanding, anastomoses are only accounted for at LV0, where the structure is reconstructed from real data. At smaller scales, the mathematical model doesn't allow such events to be taken into account. However, the brain vasculature is highly "connected": it has been shown that anastomoses may occur at all scales including at the capillary level. Indeed, it is now known that capillaries can be linked to arterial inputs through different pathways. Vascular territories thus structurally overlap, and pathways contributing the most in terms of flow will better define territories (see Blinder et al., 2013).

On a related matter, tree-like models have proven useful to describe vascular systems with fractal properties, which also concerns the cerebral intracortical vasculature, but only up to a certain resolution. At the level of capillaries, which are at the crossroads of arterial inputs and venous outputs, and are the site of fluid-tissue exchanges, microvessels are highly interconnected through anastomoses. Fractal tree-like models are no longer appropriate to describe this level of microcirculation and graph structures should be used instead, allowing flow in both directions. Such architecture, which is not accounted for in this paper since the model is limited to the pial (or surface cortical) vessels, clearly impacts the flow distribution through the different territories and its adaptation to changing situations such as occlusions or pathologies. This again supports the idea that the vascular territories should not be defined structurally but rather as non constant flow domains. Perfusion MRI techniques such as ASL allow flow territory mapping (Hendrikse et al, Stroke 2004). It could be interesting to check how these would match the structural vascular territories described in here.

The authors may want to clarify what they mean by "a unique approach for vascular reconstruction in a numerical simulation of the whole-scale cerebral blood flow." The hybrid approach, which combines both realistic image-based reconstruction and mathematical modeling, is not entirely unique or new in the sense that previous studies have developed such coupled approaches, especially in the context of blood flow modeling. For instance, Bui and colleagues (2009, 2010) have used the Circle of Willis reconstructed from real imaging data as boundary conditions for CFD simulations in fractal tree models of cerebral vasculature supplied by the major arteries (ACA, MCA, PCA).

The authors show that variability in cortical folding may have an effect on "whole-level pathways". What about the variability in vascular anatomy? Only one subject was used to extract personalized vasculature from MR angiography. However, there are many variants of different types in the population (fenestrations, duplications, absent ICA, etc). It would have been interesting to investigate interindividual variability and its effect on vascular territories for instance.

In addition, it is mentioned that a different subject was used for MRI-based brain surface reconstruction and that the vascular model was "manually adjusted to that brain surface". Again, anatomical variability can be important, especially due to the presence of sulci and gyri, and a non-rigid multimodal registration may be more appropriate here.

The authors seem concerned with computational cost and processing time. Since mathematical reconstruction in each subregion is performed independently, parallel computation seems well-suited in this application. I haven't seen it mentioned throughout the manuscript. If not done already, this is something that could greatly increase the processing speed and that might be worth considering.

Typographical errors or missing words :

P7/26 – 299: missing 's' in "patterns"

P10/26 – 373: (13) where does this value come from?

P11/26 - 378: "appearing" -> don't you mean "apparent"?

402: "for n in [1, 5]" missing "and n in [6, 13]", I believe? (for the second regression line). Same remark for legend of Fig 8

P12/26 – 450: I believe "Fig ??" should be Fig 9

465: "an areal space" should be "an area space". Or maybe "a surface space" would be more appropriate
