# Supplementary material for: Multiscale modeling of human cerebrovasculature: A hybrid approach using image-based geometry and a mathematical algorithm
Source: PLoS Comput Biol. 2020 Jun 22;16(6):e1007943. doi: 10.1371/journal.pcbi.1007943 (PMC7332106; doi:10.1371/journal.pcbi.1007943)
Supplement: Attachment — Submitted filename: rebuttal.pdf [file pcbi.1007943.s004.pdf]

Responses to Reviewer #1's comments

We thank the reviewer for the careful review and positive/constructive comments on the original manuscript, which are very helpful for improving our paper. We have revised the manuscript according to the respective comments, where major changes are highlighted in red color in the revised manuscript. Below are the item-by-item responses to all the comments from the reviewer.

Comments:

- *The cerebral vasculature goes beyond the surface of the cortex. As long as the microvasculature, which not only includes arterioles and venules but also capillaries, is not accounted for, I wouldn't call this a "whole-scale" model but rather a "multiscale" model. Or use something like "cerebral macrovasculature" instead of "cerebrovasculature" and "entire cerebral vasculature".*

  We have changed the manuscript title to 'Modeling of multiscale human cerebrovasculature: a hybrid approach using image-based geometry and mathematical algorithm' so as to appropriately describe this study.

- *It is stated in the "Model limitations and future improvements" that the "present formulation presents the possibility to address several types of anastomoses based on information on vascular subregions...". Can the authors be more specific?*

  For instance, by connecting the pathways entering into different vascular subregions, we can model a set of anastomoses that achieves a vasculature broadly spreading over the brain surface. The description has been added in 'Model limitation and future considerations' of the revised manuscript (p.17).

- *From my understanding, anastomoses are only accounted for at LV0, where the structure is reconstructed from real data. At smaller scales, the mathematical model doesn't allow such events to be taken into account. However, the brain vasculature is highly "connected": it has been shown that anastomoses may occur at all scales including at the capillary level. Indeed, it is now known that capillaries can be linked to arterial inputs through different pathways. Vascular territories thus structurally overlap, and pathways contributing the most in terms of flow will better define territories (see Blinder et al., 2013).*

  As the review pointed out, in this study the small-scale (mesoscale) vasculature does not involve the anastomoses due to the tree-based generation of the mathematical algorithm. Our model succeeds to form structural overlaps of the vascular territories because each vascular subregion is allowed to have different pathways generated from different arterial inputs. However, since we did not model the vascular connections via anastomoses, it is hard to develop an argument on the flow-related configuration of vascular territories. Therefore, we would not include these discussions in this paper. Nevertheless, we also consider the reviewer's suggestion is important to consider functional variabilities, so we would like to discuss it in our next study.

- *On a related matter, tree-like models have proven useful to describe vascular systems with fractal properties, which also concerns the cerebral intracortical vasculature, but only up to a certain resolution. At the level of capillaries, which are at the crossroads of arterial inputs and venous outputs, and are the site of fluid-tissue exchanges, microvessels are highly interconnected through anastomoses. Fractal tree-like models are no longer appropriate to describe this level of microcirculation and graph structures should be used instead, allowing flow in both directions. Such architecture, which is not accounted for in this paper since the model is limited to the pial (or surface cortical) vessels, clearly impacts the flow distribution through the different territories and its adaptation to changing situations such as occlusions or pathologies. This again supports the idea that the vascular territories should not be defined structurally but rather as non constant flow domains. Perfusion MRI techniques such as ASL allow flow territory mapping (Hendrikse et al, Stroke 2004). It could be interesting to check how these would match the structural vascular territories described in here.*

  Thank you for the valuable comments. As the reviewer pointed out, the present model might be hard to be straightforwardly applied for the microvascular modeling. So, we have removed the description 'extending

the present idea to the microvasculature' in the original manuscript (the conclusion section). As a further study, we have a plan to make use of the vascular subregion in the microvascular generation based on an existing model, analogous to the above-mentioned modeling for anastomoses. We believe, in addition to the vasculature modeling, blood flow analyses using the vascular model involving the anastomoses could bring reasonable discussions on the flow-based vascular territories. Now, we are trying to perform a blood flow simulation based on a network flow model using the present vascular model. We would like to discuss the comparison with the available data by the perfusion MRI in our next study.

- *The authors may want to clarify what they mean by "a unique approach for vascular reconstruction in a numerical simulation of the whole-scale cerebral blood flow." The hybrid approach, which combines both realistic image-based reconstruction and mathematical modeling, is not entirely unique or new in the sense that previous studies have developed such coupled approaches, especially in the context of blood flow modeling. For instance, Bui and colleagues (2009, 2010) have used the Circle of Willis reconstructed from real imaging data as boundary conditions for CFD simulations in fractal tree models of cerebral vasculature supplied by the major arteries (ACA, MCA, PCA).*

  This was an inaccurate description, so we have removed the description in Abstract. To introduce existing hybrid models not restricted to the brain, we have added a paragraph in the introduction section (fourth paragraph).

- *The authors show that variability in cortical folding may have an effect on "whole-level pathways". What about the variability in vascular anatomy? Only one subject was used to extract personalized vasculature from MR angiography. However, there are many variants of different types in the population (fenestrations, duplications, absent ICA, etc). It would have been interesting to investigate interindividual variability and its effect on vascular territories for instance.*

  We totally agree with the reviewer. We also think further comparisons among the models at different subject-specific data for both the brain shape and macrovasculature are needed to understand commonalities and variabilities of cerebrovascular systems. Although the present modeling is able to address these variabilities, we need more data on the subject and pathological specificities. We would elaborate on them in our next study. We have stated it in the last paragraph of the subsection 'Model limitations and future considerations' (p.17).

- *In addition, it is mentioned that a different subject was used for MRI-based brain surface reconstruction and that the vascular model was "manually adjusted to that brain surface". Again, anatomical variability can be important, especially due to the presence of sulci and gyri, and a nonrigid multimodal registration may be more appropriate here.*

  Thank you for giving the valuable information. We have added the following description in the subsection 'Model limitations and future considerations' (p.17).

  'Although we manually adjusted the positions, a sophisticated position adjustment algorithm such as a nonrigid multimodal registration is desired to keep a generality and avoid systematic errors in the modeling.'

- *The authors seem concerned with computational cost and processing time. Since mathematical reconstruction in each subregion is performed independently, parallel computation seems well-suited in this application. I haven't seen it mentioned throughout the manuscript. If not done already, this is something that could greatly increase the processing speed and that might be worth considering.*

  That is a good point. We have added the discussion on the execution time for the modeling (p.16).

  'We also remark that a parallel computing technology promises to provide a reduction of the modeling time because of the independence of the LV2 reconstruction, and this could be enhanced with a smaller N_u.'

Typographical errors or missing words:

- *P7/26 – 299: missing 's' in "patterns"*

  Revised.

- *P10/26 – 373: (13) where does this value come from?*

  This comes from the equation (15) in the revise manuscript.

- *P11/26 – 378: "appearing" → don't you mean "apparent"?*

  We have deleted 'appearing'.

- *P11/26 – 402: "for n in [1, 5]" missing "and n in [6, 13]", I believe? (for the second regression line). Same remark for legend of Fig 8*

  Revised to 'for $n \in [1, 5]$ and remains'.

- *P12/26 – 450: I believe "Fig ??" should be Fig 9*

  Revised.

- *P12/26 – 465: "an areal space" should be "an area space". Or maybe "a surface space" would be more appropriate*

  Revised to 'area space'.

Other remarks on the revision:

We have restructured the methods section according to another reviewer's suggestion. The description starts with a concept of the modeling including a related figure (Fig.1). Then, the image-based models are introduced, where more detailed descriptions and related figure (as Fig.2) have been additively introduced. Here, we describe which information is needed from the image-based model to the mathematical model.

We have published the dataset, which would replicate the present results, on a research data repository 'Zenodo': `http://doi.org/10.5281/zenodo.3707179`. The data contains for the image-based models (vascular structure, polygon mesh and SDF of brain hemispheres), mathematical models (vascular structures, pathway morphology, vascular territory) and reference values from the literatures.

Responses to Reviewer #2's comments

We thank the reviewer for the careful review and constructive comments on the original manuscript, which are very helpful for improving our paper. We have revised the manuscript according to the respective comments, where major changes are highlighted in red color in the revised manuscript. Below are the item-by-item responses to all the comments from the reviewer.

General comments:

While the topic of this study is fascinating the manuscript is very difficult to read, mainly due to the poor organization. Before being ready for publication I suggest that authors do the following:

1. *Streamline your introduction to clearly describe what are the objectives of the paper – to generate a vascular network for the brain. The introduction to the current version of the manuscript misleads the reader thinking this is a paper on cerebral autoregulation, which it is not.*

   We have revised the manuscript so that the topic 'cerebrovascular modeling' is emphasized, and we have deleted the description about the CFD study. In the revised manuscript, we have also added descriptions and references for existing vascular generation models. We would appreciate it if the reviewer confirm these revisions in Abstract, Author summary and Introduction.

2. *In the beginning of the methods section include a figure and clearly discuss the components included in the workflow. Include A: Generation of large vessel network from CT data – this section is almost absent in the current study. B: Generation of small vessel arterial and venous networks. Within the latter section, again clearly note components including a) prediction of vessel radius. I do not understand why this is determined from flow rather than using information from the image. b) clearly describe the optimality principle used to generate the network (it is still not clear to me what that is). c) describe network generation (this is currently done but it is hard to read) – it would help if authors had described the process in an algorithm environment. C: Discuss how generated networks compare to data, here include a thorough discussion of what data is available and how this data can be used for model validation.*

   We have reorganized and revised/added descriptions in the methods section according to the reviewer's suggestions. Here, we first describe an overview of the approach ('Concept of cerebrovascular modeling', p.4) with a new figure (Fig.1). Then, we describe the topics: (A) reconstruction of the CT-based vasculatures ('Image-based models for macroscale vasculature and brain surface', p.4) with a new figure (Fig.2); (B) methodological details of the mathematical model ('Mathematical model for mesoscale vasculatures', p.5); and (C) evaluation ways to compare the available data ('Data evaluation', pp.10-11). The detailed item-by-item responses for the mathematical model will be shown later.

3. *The results and discussion sections are reasonable with the exception that they lack more clear description of how results found in this study compare to data.*

   We have added the descriptions how the present data are evaluated and compared to the available data in the new subsection 'Data evaluation' (pp.10-11).

Specific comments:

- ***Introduction*** *– Remove discussion of CFD and cerebral autoregulation – this study is about network generation informed by imaging data. Instead discuss literature on this topic. The last paragraph of the introduction should clearly outline the objectives of this study. To design an algorithm to generate networks linking large vessels extracted from CT images to small vessels generated using the proposed algorithm. The goal is to generate a network that fills volume best possible (I think).*

  We removed the discussion of CFD and autoregulation (second paragraph in Introduction of the original manuscript), instead we have added descriptions for existing mathematical algorithms (third paragraph in

Introduction of the revised manuscript) and hybrid approach based on observed morphology and mathematical algorithm (fourth paragraph in Introduction of the revised manuscript).

We have revised the description on the last paragraph in Introduction so that the following contents clearly show: 'The main objective of this study is to model a multiscale human cerebrovasculature consisting of image-based macroscale model and mathematical mesoscale model, and explore the vascular pathway and morphology in the model though the comparisons with available data. The goal in the mesoscale modeling is to generate a vascular pathway in a newly developed mathematical algorithm that follows a proper blood supply to the brain tissues with minimizing the vascular volume in a generation region.'

- ***Methods*** *– The methods section should start with a figure clearly outlining the workflow. I would restructure the section starting with the sub-section discussing generation of large vessels from the CT images – this is currently described on page 8, but in a very short paragraph with too few details. This first section should refer to Fig. 4a and b with a clearer description of how each flow region are determined. Also discuss how other information available from the images are used, e.g. what is the total flow and how is it measured, how is data used to validate that the generated networks are correct.*

  We have restructured the methods section according to the reviewer's suggestion. The description starts with a concept of the modeling including a related figure (Fig.1). Then, the image-based models are introduced, where more detailed descriptions and related figure (Fig.2) have been additively introduced. Here, we describe which information is needed from the image-based model to the mathematical model. Our mathematical model does not need the flow data, but use geometric information from images (as explained later). See descriptions in subsections: 'Concept of cerebrovascular modeling' and 'Image-based models for macroscale vasculature and brain surface' in methods section (pp.4-5).

- *Before in detail describing how the graphs representing the small vessels are generated include an algorithm highlighting the steps used to generate these graphs. Here it would be useful to describe differences between arterial and venous trees.*

  In the revised manuscript, the method sections start with the description of overall concepts ('Concept of cerebrovascular modeling', p.4), in which an brief introduction of the mathematical model is included. Also, we had already mentioned a basic strategy at the beginning of the MRC algorithm description (p.5). Thus, the readers could gain preliminary information of the algorithm before detailed descriptions. Nevertheless, to make a description more clear, in the revised manuscript, we have added the algorithm highlight ('Highlight' in p.9) and a new flowchart figure (Fig.4). Here, the differences between arterial and venous trees are shown.

- *Section page 5 "Determination of edge radii on vascular trees". Line 125: This section starts by discussing fluid mechanics principles noting that fluid dynamics is assuming ideal conditions. What does that mean? Please specify in detail.*

  The discussion 'fluid mechanics principles noting that fluid dynamics is assuming ideal conditions' mean a flow field obtained by a flow network model consisting of cylindrical pipes in which the flowrate and pressure difference are related with a resistance attributed to Poiseuille's flow, where there is no flow disturbance at the bifurcation. Our previous explanation was not clear, so we have simplified the description: 'edge radii are determined by the pathway tree, and inlet and outlet flow pressures (or flowrates) through a flow network model'. Due to a restructure of the methods section, we have deleted the subsection title 'Determination of edge radii on vascular trees' in the revised manuscript. The related description is shown in a new paragraph 'Geometry-prioritized CCO model' (pp.6-9). Here, we first describe the optimization way in adding a new terminal vertex, and then we describe the way to determine the edge radii. The discussion is located to a latter part of this paragraph (p.9).

- *Please specify how flow is obtained from 4D-CT images – needed to understand why the radius is predicted as a function of flow – why not use information on radius from the images?*

- *The paragraph on page 6 lines 160-172 is difficult to read – so the vessel radii depends on flow, but only on the size and number of terminal ends? But equation 13 states that the radii are determined from the flow. This is confusing. What role does the data play? Vessels in the images have radii – why not use these to inform the network generation?*

  We assume the radii on vascular trees are determined by functional principles of blood flow that has been employed in existing vascular generation algorithms including the CCO model. This idea is only applied for a mathematical vasculature, and not applied for the image-based vasculature because the vessel size has been already known from the observed image. In the mathematical modeling, to employ the functional principles, we temporally introduce a (inlet) flowrate in the process of formulation, but final formulation in our geometry-prioritized CCO model does not need the (inlet) flowrate information, but instead requires the radii of root vessels. So, we only make use of the root vessel radius from the image-based model in the mathematical modeling as an input. Thus, we do not need flow information from the 4D-CT images and the images are only used for reconstruction of vessel geometry for the image-based model. We have shown these discussions before the detailed description of edge-radii determination (above equation (8)) in the revised manuscript.

- *Page 6 – "generation of a new bifurcation". This paragraph mentions that the objective is to minimize volume – that information should have been introduced much earlier.*

  In the revised manuscript, we first describe the optimization principle in the paragraph 'Geometry-prioritized CCO model' (p.6) that the total intravascular volume is minimized. Also, we have mentioned the minimization objective on the last paragraph in Introduction. See the related parts.

- *Page 7 – multilevel reconstruction. Are the networks generated starting at the root reaching a specific vascular subregion or are they just branching from the root until the entire domain is filled? That is not clear. Also, it is not clear if network construction is continued until a total number of vessels are included or if it is terminated when the vessels reach a certain size.*

  The networks generated starting at the root (always) reach a specific vascular subregion. In our algorithm, a vertex within a randomly chosen vascular subregion is added as a terminal end of one of the existing networks generated from multiple roots, and it is continued until the terminal ends reach a total number we set. The generation is independently performed in each reconstruction level based on given vascular subregions and multiple roots. We have added the description in the paragraph 'Multilevel reconstruction' (p.9).

- *Page 8 – Table 1 mostly summarizes results and should therefore be referenced in the results section. Maybe the paragraph on "model parameters" really should be moved to the results section.*

  We have moved the subsection 'Model parameters' including the Table 1 to the beginning of the results section.

- *Page 9 – The first paragraph on page 9 discuss generation of arterial and venous trees and discuss that to include vascular variation each terminal subregion does not have the same number of vessels, this is also illustrated in Fig. 1, yet in Fig.6 authors note that the network shown here has exactly two arterial vessels and one vein – this is contradicting.*

  In this paper, the number of terminal edges per vascular subregion is only varied for artery in LV1 reconstruction. It is shown in Table 1 as *Allowable number of terminals per subregion* (of the revised manuscript). In Fig.6, we just visualize the pathways, in which two terminal arteries and one terminal vein enter into a vascular subregion in LV1. Thus, other patterns that the terminal arteries are varied in a range from 1 to 6 exist in the LV1 model at the present model parameter. In this paper, in order to keep the generality, we do not fix the number of terminals per vascular subregion in the description of the MRC algorithm and in Fig.3(a) of the revised manuscript (Fig.1 in the original manuscript).

- *Page 10 – discuss results shown in Fig. 7 – it would be nice if authors have shown from where in (a) the enlarged insert shown in (b) is taken. Also, here authors do include (c) values from measurements. Are these from the same data, if so in the "data" part of the methods section, that I think should be placed first, authors should describe how this statistic was gathered.*

  We revised the figure according to the reviewer's suggestion. The data for the present model is obtained from the terminal edges in LV2 models for artery and vein, respectively. The measurement data were directly extracted from the values shown in the literature. See the last subsection 'Data evaluation' in the methods section and Fig.7 in the revised manuscript.

- *Page 11 – Authors discuss ordering, using Strahler's method – but the method is not given in the methods section – please add that. On Figure 8 it would be useful to superimpose values from literature to visualize how well the generated network match measurements, even if they are not from human networks.*

  We have added the explanation of the diameter-defined Strahler order in the subsection 'Data evaluation' in the method sections. Also, the fitting lines for the rat observation from the literature are superimposed in the graph. See p.11 and Fig.8(b) in the revised manuscript.

- *Page 12 – Vascular territories – do the networks generated encode specific vascular territories a priori or do they emerge as part of the branching strategy. Please elaborate.*

  The networks emerge as part of the branching strategy. This description is added in the second paragraph of 'Vascular territories of the major cerebral arteries' in the results section (p.14).

- *Page 12 – line 450. One of the Fig is referenced as ??.*

  Revised.

- *Page 14 – Either in addition to Fig. 11 or as a new figure – it would be interesting if authors could elaborate on the effect on brain shape, e.g. by showing simulations from brains of different shapes, this could be expanded to show modification in brains with tumors or infarcts.*

  Thank you for the interesting suggestion. We also think further comparisons among the models at different subject-specific data for both the brain shape and macrovasculature are needed to understand commonalities and variabilities of cerebrovascular systems. Although the present model is able to address these variabilities, we need more data on the subject and pathological specificities. We would elaborate on them in our next study. We have stated it in the last paragraph of the subsection 'Model limitations and future considerations' (p.17).

Other remarks on the revision:

We have changed a manuscript title so as to appropriately describes this study. According to another reviewer's comment, the word of 'whole-scale vasculature' is changed to 'multiscale vasculature'.

We have published the dataset, which would replicate the present results, on a research data repository 'Zenodo': `http://doi.org/10.5281/zenodo.3707179`. The data contains for the image-based models (vascular structure, polygon mesh and SDF of brain hemispheres), mathematical models (vascular structures, pathway morphology, vascular territory) and reference values from the literatures.
