# Supplementary material for: Multiscale modeling of human cerebrovasculature: A hybrid approach using image-based geometry and a mathematical algorithm
Source: PLoS Comput Biol. 2020 Jun 22;16(6):e1007943. doi: 10.1371/journal.pcbi.1007943 (PMC7332106; doi:10.1371/journal.pcbi.1007943)
Supplement: Attachment — Submitted filename: rebuttal_auresp_2.pdf [file pcbi.1007943.s005.pdf]

Responses to Reviewer #1's comments

We thank the reviewer for the careful review and constructive comments. We have revised the manuscript according to the respective comments, where major changes are highlighted in red color in the revised manuscript. Below are the item-by-item responses to all the comments from the reviewer.

Comments:

Authors have significantly improved the manuscript, the manuscript has a number of good components, but it still lacks a clear description of the study presented. As far as I see the main message in this manuscript is Figure 5 illustrating that it is possible to reconstruct the cerebrovascular network, in which the large vessels are informed by imaging data while the smaller vessels are generated using approximation techniques. I think this paper should be presented as a method paper discussing how best to approximate the small vessels not captured by the imaging data. To convey this story I suggest to include the following changes:

- *Abstract: Remove the first paragraph - start by noting that cerebral vasculature is complex and with standard imaging techniques it is difficult to segment all vessels in the network, and that this study proposes a method to obtain these networks by explicitly segmenting the large vessels combined with approximation techniques for small vessels. The next few sentences should address how the method proposed here is validated, and what are its limitations.*

  We have removed the first paragraph in the previous manuscript and revised the manuscript according to the reviewer's suggestion. Since the model limitations in this paper have a side as a future improvement/consideration, so we do not include them here.

- *Introduction: Relate the method proposed here to other segmentation studies extracting networks from imaging data. Motivate what you intent to do with the segmented network - compute flow, couple it with a model for cerebral autoregulation, etc etc. But this would be future work as it is not included in the current study.*

  We have added the descriptions for relation with other segmentation studies and motivation related to the blood flow in Introduction (p.2, l.20-22; p.3, l.82-84).

- *Methods: I do not like the word "modeling" as this is really "network reconstruction". I suggest to take out the word modeling instead generating a title e.g. "Network Generation". Moreover, I suggest removing the first paragraph page 4 lines (88-102)*

  We consider the "network generation" is just a side of vascular modeling, where many aspects are involved in the modeling such as edge size, 3-D configuration, topological connectivity (network) and so on. Therefore, we would like to continue to use "model(ing)" in this study. In this regard, we would also like to leave the first paragraph in the method section.

- *Close to figure 2 - add more details describing how the image was segmented and the network generated for the large vessels. Include a description of how the vessels in the network are extracted from the image and connected. Also describe what quantities are stored , e.g. vessel length, vessel diameter?*

  We have added the following description in the revised manuscript (p.4, l.121-133).

  "For the image processing, a threshold-based region-growing method was used to segment the vessel regions. This method selects a seed pixel from a region of images in which a main vessel appears in an arbitrary cross-section, and performs 3-D region growing according to the minimum and maximum thresholds set for the gray values of the images. Skeletonization is then performed through the following processes: (a) a map of the distances from the boundaries of the segmented regions is formed; (b) the segmented voxels are thinned based on the maximum value of the distance map; and (c) the centerlines and vessel radii (evaluated from the distance map) are extracted with the thinned voxels. The edge centerlines are given as a set of discrete points with the coordinates and radii, and the edge connectivity (which edge is connected to the others) is

determined. Thus, we can evaluate the vessel length and radius and the connectivity of the vascular network. The details are shown in the Amira user guide."

- *Page 4: Rename the section on "preparation for mathematical modeling" the title does not describe what is "modelleld" I think the authors are describing that the next step needed to generate a network is to extract the volume (space) in which the network lies. Therefore, use a title e.g. Extracting Brain Volume (or volume enclosed by the network).*

  Our vascular generation algorithm (in the mathematical model(ing)) needs several information from the image-based geometries, not only for the brain volume, but also the vascular subregions on the brain surface, brain signed distance function in 3-D domain (for the edge modification), and extended root vessels. So, we changed the title to "Data preparation for the mathematical modeling of mesoscale vasculatures" (p.5, l.143).

- *Page 5: Delete section "Inputs to mathematical modeling"*

  Deleted.

- *Page 5: Change title "Mathematical model for mesocale vasculature" to "Construction of mesocale part of the network" or something like that.*

  Again, we think the "model" is appropriate word in this study. Nevertheless, to clarify the meaning, we have slightly changed the title to "Mathematical modeling of mesoscale vasculatures" (p.5, l.164).

- *Page 5: Here you discuss "potential features" focus on addressing features included in this study, e.g. you do not show any flow computations so remove lines 167-168*

  Instead of eliminating the related part, we changed "Blood supplies to local regions" to "Local vessel densities" (p.5, l.181).

  In our MRC algorithm, although two introduced assumptions (equivalent WSS and vascular subregion area-dependent flowrate on terminal edges) eliminate an explicit expression of the flow in the network reconstruction, the flow can be evaluated by equation (12). However, the present flow distribution is not complete because an actual flow distribution will be determined in a closed vascular loop in which the arterial and venous systems are connected, and thus we do not show the flow distribution in this study to avoid misunderstandings.

- *Page 5: Title "Definition of vascular pathways and structures" change this to generation of vascular pathways and structures.*

  In this paragraph, we first define the definition of vascular networks to explain a vascular generation algorithm. Therefore, we leave the same title (p.6, l.188).

- *Page 7: Authors discuss how the vessel radii are evaluated by studying flow in the network, but no simulations are provided showing the flow. Either take the flow discussion out or show flow calculations.*

  As the above discussion, the present MRC algorithm evaluates the flow distribution in the network, so we leave the flow discussion remained (p.7, l.258-263).

- *Page 10: Should the title of this section be "Network Validation" or something along these lines. For each evaluation type compare predicted network to data. Currently this section reads as you are calculating several metrics, but I don't see how they are related to data.*

We changed the title to "Evaluation metrics on the mesoscale vasculatures" (p.10, l.360). The metrics are referred in the results/discussion section properly, so we think the reader can find where they are related to data. We again checked the manuscript and revised the redundant expression to make it clearer.

- *Page 11: Separate the results (showing the generated networks and how they compare to data) and discussion addressing how the technique presented here compare with other studies.*

  We have revised the results/discussion so that each of them becomes clearer following the reviewer's comment. However, the subsection "Effects of coarse-scale vascular subregions on vascular structures" is difficult to be separated because a parameter dependency of N_u on several evaluation metrics resulting in a broad argument. Instead, we have separated the description to several paragraphs per topic.

- *See e.g. the study by Meijs et al. Scientific Reports 2017 DOI:10.1038/s41598-017-15617-w*

  Thank you for the valuable information. We have read the paper and added it as a reference [11].

Responses to Reviewer #2's comments

We thank the reviewer for the careful review and constructive comments. We have revised the manuscript according to the respective comments, where major changes are highlighted in red color in the revised manuscript. Below are the item-by-item responses to all the comments from the reviewer.

Comments:

I would like to thank the authors, who made a substantial effort to improve their paper and to respond to the reviewers' comments. Indeed, most of the revisions and significant issues raised appear to have been reasonably addressed.

- *However, I encourage the authors to carefully review the manuscript again, as I have identified a number of minor errors (typographical, spelling or grammatical) that would require attention, especially in the added paragraphs.*

  We apologize for our insufficient corrections. The English proofreading by a professional company has been performed for the revised manuscript, especially focusing on the new paragraph.

- *Also, even though it may seem like a detail, I would like to point out one formulation issue that caught my attention, as it appears several times in the manuscript, including in the title: "multiscale model(ing) of human cerebrovasculature" would be more correct than "model(ing) of multiscale human cerebrovasculature", in my opinion.*

  In accordance with the reviewer's comment, we have revised the expression from "model(ing) of multiscale cerebrovasculature" to "multiscale model(ing) of cerebrovasculature" for the related parts.
